# Editorial: Creativity and creativity awareness: new directions

**DOI:** 10.3389/fpsyg.2025.1710493

**Published:** 2025-10-27

**Authors:** William M. Bart, Iclal Can

**Affiliations:** ^1^Department of Educational Psychology, University of Minnesota Twin Cities, Minneapolis, MN, United States; ^2^Guidance and Psychological Counseling Program, Middle East Technical University Northern Cyprus Campus, Güzelyurt, Türkiye

**Keywords:** creativity, creativity awareness, creativity enhancement, creative thinking, creative problem solving, creative flourishing

The Borghese Gallery in Rome is home to a captivating sculpture by Gian Lorenzo Bernini. The sculpture is a life-sized portrayal of the youthful god Apollo in pursuit of the nymph Daphne who is turning into a laurel tree. It is amazing that Bernini could transform a block of marble into a light, vibrant interplay between two youths. Viewing the sculpture of Apollo and Daphne allows one to appreciate creativity.

However, not all instances of creativity are as obvious and evident as Apollo and Daphne. People vary in their awareness of creativity. For example, a professional soccer coach needs to be keenly aware of soccer creativity when watching a young soccer player demonstrate innovative moves to pass a defender and score a goal. If creativity is to flourish in a community, people need to be aware of it when exposed to it.

How individuals develop creativity awareness and intentionally engage in creative processes constitutes a primary focus of this *Frontiers in Psychology* Research Topic “*Creativity and creativity awareness: new directions*.” Readers will explore this topic with the aid of seven articles edited by Bart and Can.

In their empirical article entitled “Direction and contextual influences on creative thinking,” Tadik et al. defined discretion as a specific form of creativity awareness with a focus on how individuals intentionally decide whether to express creative ideas depending on social contexts. The authors utilized a novel method to assess the role of discretion in the creative process, and operationalized it as deliberate engagement in idea expression. By examining discretion across playful vs. work-oriented settings, their research suggests that contextual awareness influences when and how individuals engage in creative behavior as well as how they exercise discretion in producing ideas.

In an original research article entitled “Exploring creative thinking skills in PISA: an ecological perspective on high-performing countries,” Gelmez-Burakgazi and Reiss investigated how educational contexts and ecological factors shape creative thinking among students in different countries. They examined the 2022 PISA assessment of creative thinking, which assessed the ability of 15-year-old students to generate, evaluate, and refine ideas. Focusing on Singapore, Canada, and Finland — three high-performing countries — they analyzed the systemic influences on shaping student creative thinking by using Bronfenbrenner's ecological perspective. Their results highlight the influence of ecological systems on students' creative thinking abilities and suggest that cross-national insights from these high-performing countries can guide researchers, policymakers, and educators in nurturing creativity.

In an article entitled “A new perspective on scoring children's originality: a standards-based criterion-referenced assessment approach,” Bahar and Maker advanced the understanding of creativity awareness by proposing a standards-based criterion-referenced assessment approach for scoring originality with a particular focus on teaching for creativity. They illustrated their perspective with examples drawn from evidence-based educational models that are aimed at developing students' ability to solve real-world problems creatively. The authors contended that appropriate assessment methods are crucial for supporting and tracking learning and that their approach has important implications for both instructional practice and future research.

In a conceptual article entitled “Observation: unlocking, assessing, and nurturing creative problem solving,” Maker and Bahar highlighted the importance of observing students' actions to unlock, assess, and nurture creative problem solving. Drawing on 37 years of research using the DISCOVER (Discovering Intellectual Skills and Capability while Observing Varied Ethnic Responses) model, they identified observable behaviors as indicators of creative problem-solving ability. The authors also offer practical methods by describing activities, experiences, and materials that can be used to elicit and develop these skills.

In the original research article entitled “Development and validation of the scale of aesthetics and creativity in chess,” Scherbakova et al. deepened our understanding of how creative potential can be recognized and measured in specific contexts by showing how creativity and aesthetics manifest differently across levels of expertise. The authors investigated the psychometric properties of the Scale of Aesthetics and Creativity in Chess (SACC) using data from 132 chess players ranging from expert to non-expert players. Results from the Many-facet Rasch model showed that the scale was unidimensional, explaining 50.59% of the variance. The reliability indices were strong for both items (0.83) and participants (0.93). Their findings also demonstrated that the level of chess player expertise predicted SACC scores. However, Intermediate players scored lower than Beginners, Advanced, and Expert players.

Artificial intelligence is currently a popular topic in cognition, and this compendium on creativity awareness includes an article on this topic. In an empirical article titled “The influence of generative artificial intelligence on creative cognition of design students: a chain mediation model of self-efficacy and anxiety,” Hwang and Wu examined how generative AI can promote the cognition of students in design through the mediating roles of self-efficacy and anxiety reduction. Using survey data from 121 university students in Southern China, the authors found that generative AI positively influenced students' innovative thinking, as mediated by self-efficacy and anxiety reduction, including a serial mediation effect. Their results suggest that integrating AI into design curricula could promote creative cognition, improve academic achievement, and develop designer capabilities.

The seventh and final article in this Research Topic is titled “A systems approach to creative flourishing: conceptual foundations and implications for development.” In this review article, Glass introduced the concept of creative flourishing. Defined as the convergence of creative agency, creative self-efficacy, and flow proneness within a supportive environment, creative flourishing reflects the harmonious alignment of creative desire, perceived skill, and contextual experience. Glass examines intervention strategies that promote awareness of creative flourishing and discusses ways to assess its growth.

Overall, these studies have practical implications for policymakers, educators, and researchers. By presenting the dynamic interplay among individual abilities, social context, and technological innovations, this collection posits that promoting creativity in the 21st century requires an integrated and multidimensional approach. In doing so, these contributions can help recognize and nurture the creative potential in diverse contexts.

